# Liposomal Formulation of ChimeraT, a Multiple T-Cell Epitope-Containing Recombinant Protein, Is a Candidate Vaccine for Human Visceral Leishmaniasis

**DOI:** 10.3390/vaccines8020289

**Published:** 2020-06-09

**Authors:** Daniela P. Lage, Patrícia A.F. Ribeiro, Daniel S. Dias, Débora V.C. Mendonça, Fernanda F. Ramos, Lívia M. Carvalho, Bethina T. Steiner, Grasiele S.V. Tavares, Vívian T. Martins, Amanda S. Machado, João A. Oliveira-da-Silva, Thaís T.O. Santos, Camila S. Freitas, Jamil S. Oliveira, Bruno M. Roatt, Ricardo A. Machado-de-Ávila, Maria V. Humbert, Myron Christodoulides, Eduardo A.F. Coelho

**Affiliations:** 1 Programa de Pós-Graduação em Ciências da Saúde: Infectologia e Medicina Tropical, Faculdade de Medicina, Universidade Federal de Minas Gerais, Belo Horizonte, Minas Gerais 30130-100, Brazil; danipagliara@hotmail.com (D.P.L.); patty-fernandes@hotmail.com (P.A.F.R.); daniel-sdias@hotmail.com (D.S.D.); debs.mendonca@gmail.com (D.V.C.M.); fe.fonsecaramos@gmail.com (F.F.R.); grasysv@hotmail.com (G.S.V.T.); viviantamietti@yahoo.com.br (V.T.M.); manda_sanchez92@hotmail.com (A.S.M.); joaoaosilva@gmail.com (J.A.O.-d.-S.); thaisteoli@gmail.com (T.T.O.S.); camilasimoesf@gmail.com (C.S.F.); eduardoferrazcoelho@yahoo.com.br (E.A.F.C.); 2 Laboratório de Imunopatologia, Núcleo de Pesquisas em Ciências Biológicas/NUPEB, Departamento de Ciências Biológicas, Insituto de Ciências Exatas e Biológicas, Universidade Federal de Ouro Preto, Ouro Preto, Minas Gerais 35400-000, Brazil; livinha_mendes@yahoo.com.br (L.M.C.); bmroatt@gmail.com (B.M.R.); 3 Departamento de Bioquímica e Imunologia, Instituto de Ciências Biológicas, Universidade Federal de Minas Gerais, Belo Horizonte, Minas Gerais 31270-901, Brazil; bethina@unesc.net (B.T.S.); r_andrez@yahoo.com.br (R.A.M.-d.-Á.); 4 Programa de Pós-Graduação em Ciências da Saúde, Universidade do Extremo Sul Catarinense, Criciúma, Santa Catarina 88806-000, Brazil; jamil_lefqp@yahoo.com.br; 5 Neisseria Research Group, Molecular Microbiology, School of Clinical and Experimental Sciences, University of Southampton Faculty of Medicine, Southampton General Hospital, Southampton SO16 6YD, UK; M.V.Humbert@soton.ac.uk

**Keywords:** ChimeraT, visceral leishmaniasis, vaccine, liposome, saponin, Th1-type immunity

## Abstract

*Background*: Leishmaniases are neglected diseases caused by infection with *Leishmania* parasites and there are no human vaccines in use routinely. The purpose of this study was to examine the immunogenicity of ChimeraT, a novel synthetic recombinant vaccine against visceral leishmaniasis (VL), incorporated into a human-compatible liposome formulation. *Methods*: BALB/c mice were immunized subcutaneously with ChimeraT/liposome vaccine, ChimeraT/saponin adjuvant, or ChimeraT/saline and immune responses examined in vitro and in vivo. *Results*: Immunization with the ChimeraT/liposome formulation induced a polarized Th1-type response and significant protection against *L. infantum* infection. ChimeraT/liposome vaccine stimulated significantly high levels of interferon (IFN)-γ, interleukin (IL)-12, and granulocyte macrophage-colony stimulating factor (GM-CSF) cytokines by both CD4 and CD8 T-cells, with correspondingly lower levels of IL-4 and IL-10 cytokines. Induced antibodies were predominantly IgG2a isotype, and homologous antigen-stimulated spleen cells produced significant nitrite as a proxy for nitric oxide (NO). Furthermore, we examined a small number of treated VL patients and found higher levels of circulating anti-ChimeraT protein IgG2 antibodies, compared to IgG1 levels. *Conclusions*: Overall, the liposomal formulation of ChimeraT induced a protective Th1-type immune response and thus could be considered in future studies as a vaccine candidate against human VL.

## 1. Introduction

Leishmaniases are neglected diseases caused by parasites of the genus *Leishmania*. This disease complex is endemic in 98 countries and 380 million people are exposed annually to the risks of infection [1]. The clinical manifestations of disease vary from self-limiting cutaneous lesions to acute systemic visceral disease, which can cause the death of patients, if untreated [2]. Visceral leishmaniasis (VL) is second in mortality and fourth in morbidity among the tropical diseases worldwide [1,3].

Treatment against VL depends mainly on endovenous or intramuscular administration of pentavalent antimonials, which patients find uncomfortable [4]. Moreover, antimonials are toxic, and they can cause chemical pancreatitis, increases in serum aminotransferase levels, and electrocardiographic abnormalities. The development of parasitic resistance to pentavalent antimonials has also been reported [5]. Amphotericin B (AmpB) is another effective antileishmanial drug, but it is similarly toxic to mammalian hosts [6]. AmpB-based liposomal formulations reduce drug toxicity whilst maintaining their efficacy against *Leishmania*, but these formulations are expensive [7,8]. Alternative therapeutic agents include miltefosine, which was originally developed as an anticancer drug [9]. Miltefosine is the first drug used orally for treating VL patients [10,11], but it can cause teratogenicity, and parasite resistance has also been reported [12,13], which also impacts on its clinical use. The development of a prophylactic human vaccine against VL is strongly considered as a non-toxic, safer, and low-cost alternative to control the spread of disease worldwide.

Immunity against *Leishmania* infection in mammalian models depends on the development of a Th1-type immune response, characterized by the production of high levels of IFN-γ, IL-12, IL-2, among other proinflammatory cytokines. These and other molecules stimulate infected macrophages to eliminate internalized parasites and protect against the development of disease [14]. Conversely, Th2-type cells produce anti-inflammatory cytokines, e.g., IL-4, IL-10, and IL-13, which deactivate parasitized macrophages and contribute to disease development [15]. The humoral response during human VL has also been studied, and patients with active disease usually have high levels of anti-*Leishmania* IgG antibodies [16,17,18,19]. In addition, IL-10 is known to promote B cell proliferation and the IgG isotype switch toward IgG1 and IgG3 [20]. High IgG1 antibody titers found in active VL could indicate a progressive decline in the immune status of patients and consequently to more severe disease. By contrast, IFN-γ is known to induce an increase in anti-*Leishmania* IgG2 isotype levels, which are found in higher levels in treated and cured patients [21].

Despite substantial knowledge of the parasite genome and proteome, developing a human VL vaccine continues to be a significant challenge [22]. From the proteome/immuno-proteome, several protein antigens have been examined and good results have been obtained in murine and/or canine models [23,24,25,26], but these have not been translated to human studies [27]. Recent studies have shown that multiple epitope-based vaccines can induce more potent immune responses than the use of individual protein vaccines, since T-cells from genetically distinct populations would recognize and respond to a single peptide epitope. This observation underlines the need to identify more specific T-cell epitopes that bind to multiple Human Leukocyte Antigen (HLA) alleles and could cover almost 100% of the human population [28,29,30].

Most protein-based vaccines fail to induce significant cellular responses without the use of adjuvants [31] and experimental VL vaccines are no exception [32,33]. Furthermore, adjuvants are necessary to increase the duration of protection and/or reduce the number of vaccine doses [34]. Previous studies have shown that saponins, which are glycosides capable of stimulating T-cells to produce Th1-type cytokines [35], have been successful as immuno-adjuvants with recombinant *Leishmania* proteins [36,37,38,39,40,41], chimeras [27,30], and synthetic peptides [42,43]. Although saponins are used in mice and dogs, they are no longer recommended for human use, since they can cause granulomatous reactions, hemolysis, local pain, and other adverse reactions. Thus, alternative safe and effective human-compatible adjuvants and/or delivery vehicles need to be evaluated with candidate *Leishmania* vaccines. Liposomes are a primary choice of adjuvant-delivery vehicle, as they are biodegradable, non-toxic, and can stimulate Th1-type immune responses [22,44]. They have been used for drug delivery and as immuno-adjuvants, e.g., against cancer [45], diarrheal disease [46], human immunodeficiency virus infection [47], and many others. Thus, liposomes present no safety issues for human use.

Recently, we designed and developed a synthetic recombinant vaccine called ChimeraT, which contains specific T-cell epitopes from the *Leishmania* prohibitin, eukaryotic initiation factor 5a, and hypothetical LiHyp1 and LiHyp2 proteins (Lage et al., manuscript submitted). The rationale for choosing these four proteins to develop ChimeraT is based on previous studies that have shown them to be antigenic and immunogenic, and by the fact that they were recognized by antibodies from humans developing VL [48,49,50,51,52]. This recognition by the immune system of infected hosts during active disease suggests that these proteins could have vaccine potential. Importantly, our recent study (Lage et al., manuscript submitted) showed that the protective activity of ChimeraT was superior in direct comparison with the individual recombinant proteins. Generation of multiple T-cell epitope ChimeraT vaccines offers several advantages over vaccines using single or combinations of recombinant proteins. For example, chimera vaccines can be tailored to contain the most antigenic CD4^+^ and CD8^+^ T-cell epitopes from distinct immunogenic *Leishmania* proteins, which avoids epitopes present in whole proteins with low immunogenicity or undesirable immunodominance [26]. Chimera vaccine designs allow the potential to further rationally engineer the epitopes to increase their immunological potency [27,53]; they could improve safety by reducing antigen load, reduce antigenic competition that may occur with the use of individual recombinant proteins, and reduce the cost of manufacture and enable more facile quality control.

ChimeraT delivered with saponin as the adjuvant induced a Th1-type immune response and protected BALB/c mice against *L. infantum* infection (Lage et al., manuscript submitted). In the present study, we report that a ChimeraT/Liposome formulation induces a potent Th1-type protective immune response in mice and is preferred to saponin, thus providing support for translation to studies in humans.

## 2. Materials and Methods

### 2.1. Production of Recombinant ChimeraT Protein

To produce the recombinant protein ChimeraT, the amino acid sequences of the *Leishmania* hypothetical protein 1 (LiHyp1, XP_001468941.1), *Leishmania* hypothetical protein 2 (LiHyp2, XP_001462854.1), prohibitin (PHB, XP_001468827.1), and eukaryotic initiation factor 5a (EIF5a, XP_001466105.1) proteins were analyzed with bioinformatics tools to select regions containing immunogenic epitopes that bind specifically to murine and human T-cell haplotypes. T-cell epitopes were predicted by the Rankpep server (imed.med.ucm.es/Tools/rankpep.html) [54], with the H-2Db, H-2Dd, H-2Kb, H-2Kd, H-2Kk, and H-2Ld alleles from mouse and A2, A3, A24 and B7 alleles from human used to predict the CD8^+^ T-cell epitopes, and the H-2IAb, H-2IAd, H-2Ias, H-2IEd, and H-2IEb alleles from mouse and HLA-DR alleles from human were used to predict the CD4^+^ T-cell epitopes. The following T-cell epitopes were selected: PHB (104-VRVLYQPNVENLYHIYRHIGVNAETVL-131), EIF5a (71-RLEDQAPSTHNVEVPFVKTFTYSVLDIQPNE-101), LiHyp1 (163-YIMSGPARYVYFHMVLPVEAQ-183), and LiHyp2 (416-NYDPNVWCAVPNCITCDRLDPSNR-439).

To design the chimeric protein-encoding gene, the amino acids of each epitope were translated into the corresponding DNA coding sequence using the Standard Genetic Code Map. After conversion, the sequences were grouped linearly with two glycine residues as spacers between them to create a single protein. The position of each epitope within the protein sequence was chosen to mimic their arrangement in their own source protein. Therefore, the epitopes were ordered as follows: LiHyp1 epitope–GG–EIF5a epitope–GG–LiHyp2 epitope–GG–PHB epitope. Codon optimization for expression in *Escherichia coli* was done in silico with the web Codon Optimization tool (https://www.idtdna.com/CodonOpt) and optimized to reduce the presence of intramolecular interactions of mRNA, which were calculated by using the MFOLD Program. The physicochemical characteristics of the ChimeraT and the individual T-cell epitopes were calculated with the ProtParam tool [55]. The ChimeraT-encoding gene was cloned commercially in the pET-28a (+)-TEV vector (Genscript^®^, USA). Expression of the recombinant protein has been described recently (Lage et al., manuscript submitted). Briefly, ChimeraT was expressed in *E. coli* DE3 cells (Artic Express Agilent Technologies, USA) after induction with 1 mM isopropyl β-D-1-thiogalactopyranoside (IPTG) and purified by HisTrap HP affinity chromatography followed by Superdex^TM^ 200 gel-filtration. After purification, the recombinant ChimeraT protein was passed through a polymyxin-agarose column to remove any residual endotoxin (<10 ng of lipopolysaccharide per 1 mg of ChimeraT protein was quantified with the Quantitative Chromogenic Limulus Amebocyte Assay QCL-1000; BioWhittaker, USA). The physiochemical properties of ChimeraT (109 amino acids, relative molecular mass (*Mr*) 12.3 kDa) have been described in detail recently (Lage et al., manuscript submitted).

### 2.2. Preparation of Liposomes Incorporating ChimeraT

Liposomes were prepared as described previously [33]. Briefly, chloroform solutions containing 1,2-dipalmitoyl-sn-3-phosphocholine (DPPC; Lipoid GmbH Ludwigshafen, Germany) and cholesterol (CHOL; Sigma-Aldrich, USA) were prepared, and aliquots (total lipid concentration equal to 10 mM at a 6:4 molar ratio of DPPC:CHOL) were transferred to a round-bottom flask and evaporated under reduced pressure at 30 °C (rotary evaporator R-215 and vacuum pump V-700 model; Buchi Labortechnik AG, Flawil, Switzerland). The lipid film was hydrated at room temperature with phosphate-buffered saline (PBS, pH 7.4) containing ChimeraT (1.0 mg/mL) using a shaker apparatus (model MS1, Staufen, Germany). The resulting multilamellar vesicle dispersion was calibrated by extrusion (Lipex^®^ biomembrane extruder; Northern Lipids, Burnaby, BC, Canada) of the formulation through a polycarbonate membrane with a pore size of 0.4 μm (10 cycles; Millipore, Billerica, MA, USA). Liposomes were centrifuged at 300,000× *g* for 2 h at 10 °C (Optima^®^ L-80XP; Beckman Coulter, Indianapolis, IN, USA), and their mean diameter and polydispersity index were evaluated by dynamic light scattering at a 90° angle, at 25 °C. The zeta (ζ)-potential was evaluated by determining the electrophoretic mobility at a 90° angle. The samples were diluted in PBS and measurements were done in a Zetasizer Nano ZS90 apparatus (Malvern Instruments, Malvern, UK). The final ChimeraT protein concentration in the liposomal formulation was 0.83 mg/mL. Control liposomes were similarly prepared, without ChimeraT.

ChimeraT/Liposome showed an average size of 435.6 nm ± 19.5 nm, a polydispersity index of 0.58 ± 0.12, and a ζ-potential of 3.85 ± 0.48 mV. The corresponding control empty liposome preparation showed a mean diameter of 129.7 ± 16.6 nm, a polydispersity index of 0.45 ± 0.10, and a ζ-potential of 4.11 ± 0.39 mV.

### 2.3. Mice and Parasites

The study was approved by the Committee on the Ethical Handling of Research Animals (CEUA) of the Federal University of Minas Gerais (UFMG, protocol number 333/2015). Female BALB/c mice (specific pathogen free, 8 weeks of age) were obtained from the breeding facilities of the Department of Biochemistry and Immunology, Institute of Biological Sciences, UFMG (Belo Horizonte, Minas Gerais, Brazil), and maintained under specific pathogen-free conditions. *L. infantum* (MHOM/BR/1970/BH46) strain was used. Parasites were grown at 24 °C in Complete Schneider’s Medium (Sigma-Aldrich, St. Louis, MO, USA), pH 7.4, containing 20% (*v*/*v*) heat-inactivated fetal bovine serum (FBS; Sigma-Aldrich, St. Louis, MO, USA), 20 mM L-glutamine, 200 U/mL penicillin, and 100 µg/mL streptomycin. The soluble *Leishmania* antigenic (SLA) extract was prepared from *L. infantum* stationary promastigotes as described previously [36].

### 2.4. Vaccination of BALB/c Mice and Infection with L. infantum

BALB/c mice (n = 16 per group) were immunized subcutaneously in their left hind footpad with ChimeraT (20 μg) in saline or in combination with saponin (*Quillaja saponaria* bark saponin; Sigma-Aldrich, St. Louis, MO, USA) (20 μg protein plus 20 μg Saponin) or in liposome (containing 20 μg of ChimeraT protein). Control mice received saline, saponin, or empty liposome alone. The immunization schedule was three doses administered at 14-day intervals and 30 days after the third immunization, and mice (n = 8 per group) were euthanized for evaluating specific immune responses (before infection). The remaining animals (n = 8 per group) were subcutaneously infected in their right hind footpad with 10^7^ *L. infantum* stationary promastigotes and then followed for 45 days.

### 2.5. Evaluating Cellular Responses before and after L. infantum Infection

Spleen cells were collected from mice (n = 8 per group) euthanized at 30 days after the last vaccine dose (before infection) and at 45 days post-infection (after infection). Splenocytes (5 × 10^6^ cells/mL) were plated in 24-well plates (Nunc) in complete RPMI 1640 medium (non-stimulated, control) or stimulated with ChimeraT or *L. infantum* SLA (10.0 and 25.0 μg/mL, respectively) for 48 h at 37 °C in 5% (*v*/*v*) CO_2_. IFN-γ, IL-4, IL-10, IL-12p70, and GM-CSF levels were quantified in spleen cell culture supernatants by capture ELISA (BD OptEIA TM set mouse kits; Pharmingen, USA), following the manufacturer’s instructions. The presence of nitrite in cell supernatants was quantified by Griess reaction [56]. In addition, the IFN-γ-producing T-cell profile in the ChimeraT/Saponin and ChimeraT/Liposome groups was evaluated by the addition of anti-CD4 (GK 1.5) or anti-CD8 (53-6.7) monoclonal antibodies into the in vitro cultures (5 μg of each antibody; Pharmingen^®^, San Diego, CA, USA), for a period of 48 h at 37 °C with 5% CO_2_. Anti-IL-12 monoclonal antibody (C17.8; 5 μg; Pharmingen) was included as a positive control. Appropriate isotype-matched controls (rat IgG2a (R35-95) and rat IgG2b (95-1)) were used.

### 2.6. Humoral Response Generated before and after L. infantum Challenge

ChimeraT and SLA-specific IgG1 and IgG2a antibody levels were evaluated qualitatively in sera from infected and/or vaccinated mice by ELISA as described previously [30]. Briefly, titration curves were done to determine the most appropriate antigen concentration and antibody dilution to be used. Then, Falcon flexible microtiter immunoassay plates (Becton Dickinson, Franklin Lakes, NJ, USA) were coated at 4 °C for 16 h with ChimeraT or *L. infantum* SLA (0.25 and 1.0 µg per well, respectively) in 100 µL per well of coating buffer (50 mM carbonate buffer, pH 9.6). Free binding sites were blocked with 200 µL of PBS containing 0.05% (*v*/*v*) Tween 20 (PBST) and 5% (*w*/*v*) bovine serum albumin (BSA) for 1 h at 37 °C. After five washes with PBST, wells were incubated with 100 µL of sera (diluted 1/100 in PBST), for 1 h at 37 °C. Plates were washed five times with PBST and wells incubated for 1 h at 37 °C with IgG total-, IgG1-, or IgG2a-specific peroxidase-labeled antibody (Sigma-Aldrich, St. Louis, MO, USA), diluted in PBST at 1/20,000, 1/10,000, and 1/20,000, respectively. After washing five times, reactions were developed by the addition of H_2_O_2_, ortho-phenylenediamine, and citrate-phosphate buffer, pH 5.0, for 30 min in the dark and stopped by adding 2N H_2_SO_4_. Absorbance was read at λ492 nm in an ELISA microplate spectrophotometer (Molecular Devices, Spectra Max Plus, San Jose, CA, USA).

### 2.7. Evaluation of the Parasite Load by the Limiting Dilution Technique and Reverse Transcriptase-Polymerase Chain Reaction (RT-PCR)

Mice (n = 8 per group) were euthanized at 45 days after *L. infantum* infection and the spleens, livers, bone marrows, and draining lymph nodes were collected for evaluation of the parasite load by a limiting dilution technique and RT-PCR. For the limiting dilution technique [27], organs were weighed and homogenized using a glass tissue grinder in sterile PBS. Tissue debris was removed (centrifugation at 150× *g*), and cells were concentrated (centrifugation at 2000× *g*). Pellets were suspended in 1 mL of Complete Schneider’s Medium and 220-µL volumes were plated into 96-well flat-bottom microtiter plates (Nunc), and diluted serially in complete Schneider’s Insect Medium (10^−1^ to 10^−12^ dilution). Each sample was plated in triplicate, incubated at 24 °C and read 7 days later by microscopy. Results were expressed as the negative log of the titer (i.e., the dilution corresponding to the last positive well) adjusted per milligram of organ. Parasitism in the spleens of infected and vaccinated animals was also evaluated by RT-PCR [57]. Briefly, DNA was extracted using a Wizard^®^ Genomic DNA Purification Kit (Promega Corporation) and suspended in milli-Q water. Parasite load was determined using *Forward* primer 5′-CCTATTTTACACCAACCCCCAGT-3′ and *Reverse* primer 5′-GGGTAGGGGCGTTCTGCGAAA-3′ [50]. Reactions were processed in an ABI Prism 7500 Sequence Detection System (96 wells-plate; Applied Biosystems, Waltham, MA, USA), using 2× SYBR^TM^ Select Master Mix (5 µL; Applied Biosystems), 2 mM of each primer (1 µL), and 4 µL of DNA (25 ng/µL). The samples were incubated at 95 °C for 10 min, and submitted to 40 cycles of 95 °C for 15 s and 60 °C for 1 min. During each time, fluorescence data were collected. Parasite load was quantified by interpolation from the standard curve, which was done in duplicate and converted to the number of parasites per nucleated cell (multiplied by 1000 to facilitate visualization).

### 2.8. Human Sera

The study was approved by the Human Ethics Committee of UFMG (CAAE–32343114.9.0000.5149). VL patients (n = 8, including 4 males and 4 females, with ages ranging from 31 to 62 years) living in an endemic region of disease (Belo Horizonte) were diagnosed by PCR, which identifies *L. infantum* kDNA in aspirates of spleen and/or bone marrow. The patients also presented positive serological results by a Kalazar Detect™ Test Kit (InBios International, Inc., Seattle, WA, USA). Serum was collected from each patient, before and six months after treatment with pentavalent antimonials (Sanofi Aventis Farmacêutica Ltd.a., Suzano, São Paulo, Brazil).

### 2.9. Post-Therapy Serological Follow-up

Anti-ChimeraT and anti-parasite IgG1 and IgG2 antibody isotype production in VL patients, before and after treatment, was evaluated qualitatively by ELISA assay as described above. Immunoassay plates were coated with the ChimeraT or *L. infantum* SLA (0.5 and 1.0 µg per well, respectively) and sera from VL patients were all tested at 1/100 dilution in PBST.

### 2.10. Statistical Analysis

Data were input into Microsoft Excel (version 10.0) and GraphPad Prism^TM^ (version 6.0 for Windows). One-way analysis of variance (ANOVA) and Bonferroni’s test was done to compare the experimental groups, and differences were considered significant with *p* values < 0.05.

## 3. Results

### 3.1. Cellular Responses in Vaccinated Mice before and after L. infantum Infection

Initially, spleens were collected from BALB/c mice that had been vaccinated with ChimeraT/liposome, ChimeraT/saponin, ChimeraT/saline, saline, saponin, or empty liposome but were not infected with *L. infantum*. Spleen cells were cultured and stimulated with ChimeraT and SLA, in order to examine the specificity of the cytokine response to the vaccinating antigen and to the parasite protein extract, respectively [58,59,60,61,62,63]. Spleen cell supernatants were collected, and the production of selected Th1 and Th2-type cytokines was evaluated as markers of the cellular immune response and as an indicator of the potential efficacy of the immunogen against infection by the parasites [64,65,66].

**Figure 1:**
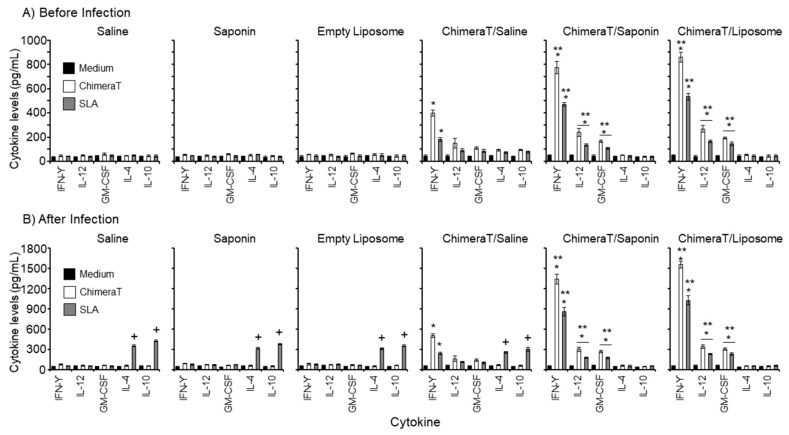
Cytokine responses. BALB/c mice (n = 8 per group) were immunized with ChimeraT, ChimeraT/saponin, or ChimeraT/liposome and controls received saline, saponin, or empty liposome. Spleens were collected 30 days after the last vaccine dose. The remaining animals (n = 8 per group) were challenged with 10^7^ *L. infantum* stationary promastigotes, and 45 days after infection, their spleens were also collected. In both cases, cultured splenocytes (5 × 10^6^ cells per mL) were stimulated with ChimeraT (10 µg/mL) or *L. infantum* SLA (50 µg/mL). IFN-γ, IL-12, GM-CSF, IL-4, and IL-10 cytokine levels were quantified by ELISA in cell supernatants. Results show the cytokine levels (**A**) before and (**B**) after infection. The columns indicate the mean ± standard deviation of the groups. (*) indicates a significant difference compared to the control saline, saponin, and empty liposome groups (*p* < 0.0001), when ChimeraT and SLA were used as stimuli as defined by the accompanying horizontal bar beneath. (**) indicates a statistically significant difference compared to the ChimeraT/saline group (*p* < 0.0001), when ChimeraT and SLA were used as stimuli as defined by the accompanying horizontal bar beneath. (+) indicates a statistically significant difference compared to the ChimeraT/saponin and ChimeraT/liposome groups (*p* < 0.0001).

Spleen cells from the ChimeraT/liposome-, ChimeraT/saponin-, and ChimeraT/saline-immunized mice, when stimulated with either ChimeraT or SLA, produced significantly higher levels of IFN-γ, IL-12, and GM-CSF cytokines than the control groups (Figure 1A). For example, spleen cells from mice immunized with ChimeraT/Saline and stimulated with ChimeraT protein secreted 9-, 3-, and 2-fold more IFN-γ, IL-12, and GM-CSF than immunization with the respective controls. With SLA as the stimulant, ~2-4-fold increases in these cytokines were observed. Spleen cells from mice immunized with ChimeraT/liposome and ChimeraT/saponin were essentially similar and higher than those observed with the ChimeraT/saline group. For example, spleen cells from the ChimeraT/liposome and ChimeraT/saponin groups produced ~16-, 5-, and 3-fold increases in IFN-γ, IL-12, and GM-CSF after stimulation with ChimeraT protein, compared to the respective control groups. With SLA as the stimulant, ~10-12-fold increases were observed for IFN-γ, and ~3-4-fold increases for IL-12 and GM-CSF, with both the ChimeraT/liposome and ChimeraT/saponin groups. No significant secretion of IL-4 and IL-10 was observed in any experimental group before infection (Figure 1A).

Spleen cells were then collected from groups of vaccinated and control mice at 45 days after infection with *L. infantum*. Similar cytokine profiles and fold increases were observed after infection (Figure 1B). For example, spleen cells from mice immunized with ChimeraT/Saline and stimulated with ChimeraT protein secreted 7-, 3-, and 2-fold more IFN-γ, IL-12, and GM-CSF than immunization with the respective controls. With SLA as the stimulant, ~2-4-fold increases in these cytokines were observed. Spleen cells from the ChimeraT/liposome and ChimeraT/saponin groups produced ~15-19-fold increases in IFN-γ and ~4-5-fold increases in IL-12 and GM-CSF after stimulation with ChimeraT protein, compared to the respective control groups. With SLA as the stimulant, ~11-14-fold increases were observed for IFN-γ, and ~3-4-fold increases for IL-12 and GM-CSF, with both ChimeraT/liposome and ChimeraT/saponin groups. By contrast, spleen cells from the saline, saponin, and empty liposome control mice produced ~7-fold significantly higher parasite (SLA)-specific IL-4 and IL-10 cytokines than spleen cells from mice vaccinated with ChimeraT/liposome or ChimeraT/saponin. However, no significant difference was observed in parasite-specific IL-4 or IL-10 cytokine levels produced by spleen cells from mice immunized with ChimeraT/saline, compared with the other control groups.

We used the data from Figure 1 to calculate the ratio of IFN-γ/IL-10 cytokines, which is known to correlate with enhanced Th-1 type immune activation. Before infection, the IFN-γ/IL-10 ratio was 4.0 for mice immunized with ChimeraT/saline and tested against ChimeraT antigen, and this increased to 20.0 for both the ChimeraT/saponin and ChimeraT/liposome groups (Table 1). With SLA as the antigen, the ratio was 2.0 for ChimeraT/saline and 12.0 for the two adjuvanted preparations (Table 1). After infection, the IFN-γ/IL-10 ratio increased to ~8.0 for mice immunized with ChimeraT/saline and tested against ChimeraT antigen and increased further to ~30.0–33.0 for both the ChimeraT/saponin and ChimeraT/liposome groups (Table 1). With SLA as the antigen, the ratio was ≤1.0 for ChimeraT/saline and 17.0–19.0 for the two adjuvanted preparations (Table 1).

**Figure 2:**
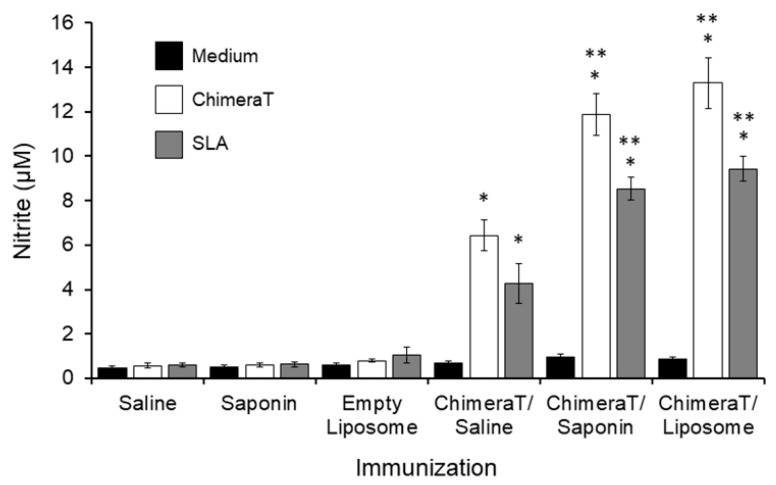
Nitrite secretion levels in the culture supernatants of vaccinated and infected mice, evaluated in vitro by the Griess method. The cell supernatants used to evaluate ChimeraT protein and parasite-specific cytokine levels after *L. infantum* infection were also used for quantifying nitrite secretion. The bars indicate the mean ± standard deviation of the groups. (*) indicates a significant difference compared to the control saline, saponin, and empty liposome groups (*p* < 0.0001). (**) indicates a statistically significant difference compared to the ChimeraT/saline group (*p* < 0.0001).

Production of nitrite as a proxy for nitric oxide (NO) was evaluated after infection (Figure 2). The rationale for this is based on the observation that the production of NO by the inducible NO synthase enzyme is believed to be a key defense mechanism against *Leishmania* infection [67]. Spleen cells from the ChimeraT/saline, ChimeraT/saponin, and ChimeraT/liposome mice stimulated with ChimeraT significantly increased nitrite production by 11-, 20-, and 17-fold compared to the respective control groups. With SLA as the stimulant, spleen cells from the ChimeraT/saline, ChimeraT/saponin, and ChimeraT/liposome mice showed 7-, 14-, and 9-fold increases in nitrite production compared to the respective control groups. There were no significant differences between the levels of nitrite production associated with the ChimeraT/liposome and ChimeraT/saponin groups and the levels were ~2-fold higher than those observed with the ChimeraT/saline group.

**Figure 3:**
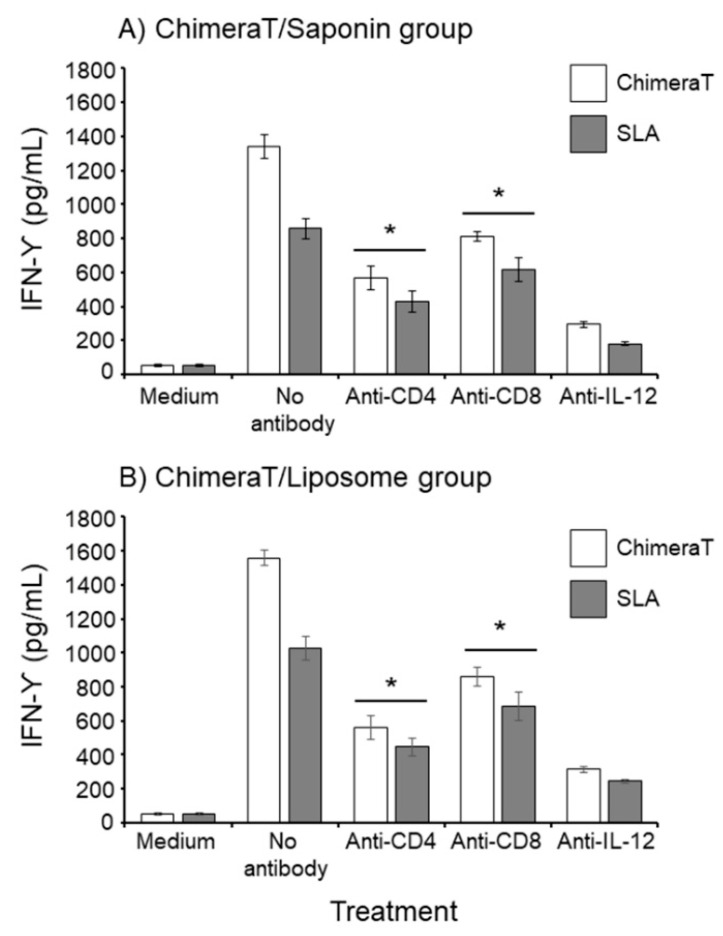
Involvement of CD4 and CD8 T-cell subtypes in IFN-γ production in the ChimeraT/liposome and ChimeraT/saponin groups after infection. Cultured splenocytes (5 × 10^6^ cells per mL) of the ChimeraT/saponin and ChimeraT/liposome groups were stimulated with ChimeraT protein or *L. infantum* SLA (10 and 50 µg/mL, respectively), in the absence (medium) or presence of anti-CD4, anti-CD8, or control anti-IL-12 monoclonal antibodies. Cultures were also maintained without monoclonal antibodies as controls. IFN-γ levels were quantified by ELISA in cell supernatants of the ChimeraT/saponin (Panel **A**) and ChimeraT/liposome groups (Panel **B**). (*) indicates the statistically significant difference compared with the respective no antibody control treatment (*p* < 0.0001), when ChimeraT and SLA were used as stimuli as defined by the accompanying horizontal bar beneath.

Next, we quantified the IFN-γ-producing T-cell profile for the spleen cells taken specifically from the best-performing groups, i.e., the ChimeraT/liposome- and ChimeraT/saponin-vaccinated mice. For the ChimeraT/liposome group, the addition of anti-CD4, anti-CD8, and control anti-IL-12 monoclonal antibodies significantly reduced IFN-γ production by ChimeraT-stimulated cells by 58.0%, 39.0%, and 78.0%, respectively, and by SLA-stimulated cells by 50.0%, 28.0%, and 79.0%, respectively, when compared to the values obtained with the no antibody controls (Figure 3). Similar results were obtained for the ChimeraT/saponin group: The addition of anti-CD4, anti-CD8, and anti-IL-12 monoclonal antibodies significantly reduced IFN-γ production by ChimeraT-stimulated cells by 64.0%, 45.0%, and 80.0%, respectively, and for SLA-stimulated spleen cells by 56.0%, 33.0%, and 76.0%, respectively, when compared to the values obtained with the no antibody controls (Figure 3).

### 3.2. Total IgG, IgG1, and IgG2a Antibody Responses in Vaccinated Mice before and after L. infantum Infection

**Figure 4:**
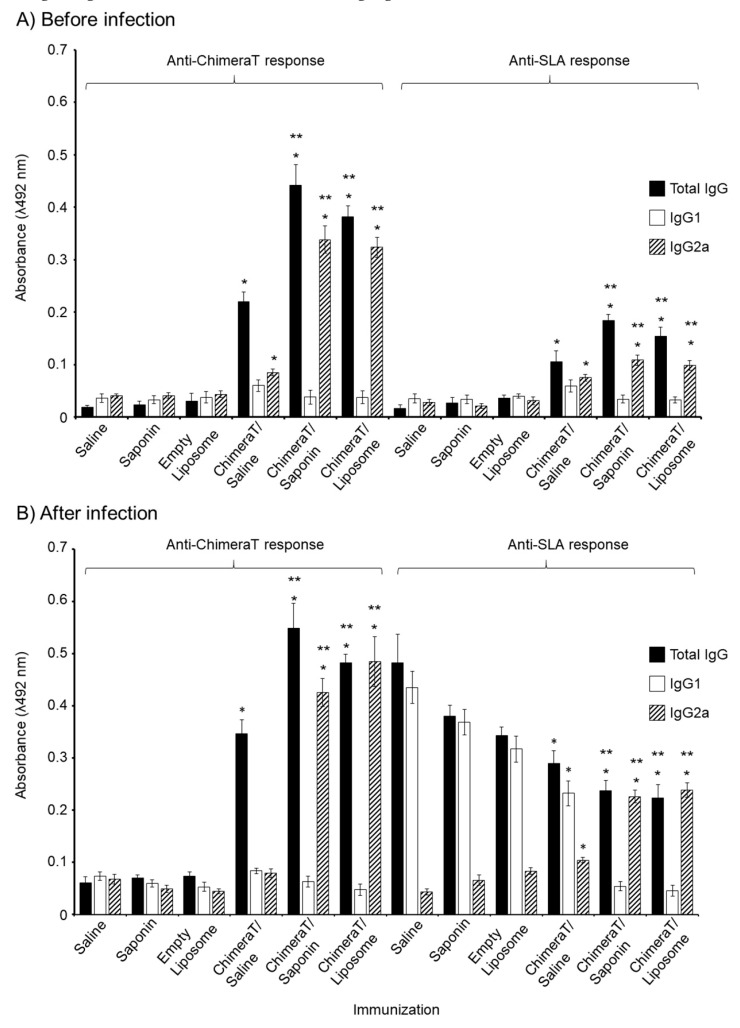
Anti-ChimeraT protein and anti-SLA humoral responses in infected and/or vaccinated mice. BALB/c mice (n = 8 per group) were immunized with ChimeraT/saline, ChimeraT/saponin, or ChimeraT/liposome and controls received saline, saponin, or empty liposome. Sera were collected at 30 days after the last vaccine dose. Mice were challenged with 10^7^ *L. infantum* stationary promastigotes and sera collected at 45 days after infection. In both cases, total IgG, IgG1, and IgG2a antibody levels specific to ChimeraT and SLA were measured qualitatively by ELISA. Results show antibody levels before (Panel **A**) and after (Panel **B**) infection. The bars indicate the mean ± standard deviation of the groups. (*) indicates significant difference compared to the control saline, saponin, and empty liposome groups (*p* < 0.0001). (**) indicates statistically significant difference compared to the ChimeraT/saline group (*p* < 0.0001).

Before infection, mice vaccinated with ChimeraT/saline, ChimeraT/saponin, and ChimeraT/liposome produced total IgG antibodies specific for ChimeraT protein and SLA, with the adjuvanted formulations inducing significantly higher IgG than ChimeraT/saline (Figure 4A). Mice immunized with ChimeraT/liposome and ChimeraT/saponin showed a significant ~9-fold ratio increase in IgG2a antibodies relative to IgG1 reacting with ChimeraT protein, which was significantly higher than the ratio of ~1.4 for sera from mice immunized with ChimeraT/saline (Figure 4A; Table 2). When measured against SLA antigen, ChimeraT/liposome and ChimeraT/saponin showed significant ~3-fold ratio increases in IgG2a antibodies relative to IgG1, whereas no difference was observed in the lower levels of IgG1 and IgG2a induced by ChimeraT/saline. None of the control mice produced IgG antibodies.

A similar humoral profile was found after infection in mice vaccinated with ChimeraT/saline, ChimeraT/saponin, and ChimeraT/liposome, with significant increases in total IgG observed to ChimeraT protein compared with the control groups (Figure 4B). Mice immunized with ChimeraT/liposome and ChimeraT/saponin showed a significant ~10- and 7-fold ratio increase, respectively, in IgG2a antibodies relative to IgG1 reacting with ChimeraT protein, whereas the lower levels of both isotypes from mice immunized with ChimeraT/saline were similar (ratio of ~1.0) (Figure 4B; Table 2). When measured against SLA antigen, ChimeraT/liposome and ChimeraT/saponin showed a significant ~4-5-fold ratio increase in IgG2a antibodies relative to IgG1, whereas conversely, there was a 2-fold ratio increase in IgG1 antibodies relative to IgG2a in sera from mice immunized with ChimeraT/saline. After infection, as expected, the control groups (saline, saponin, and empty liposome) showed significant increases in total IgG to the SLA parasite extract, compared to the levels before infection. These antibodies were predominantly of the IgG1 isotype, with the IgG1/IgG2a ratios for these controls ranging from ~4 to 10 (Table 2).

### 3.3. Estimation of Parasite Burden

**Figure 5:**
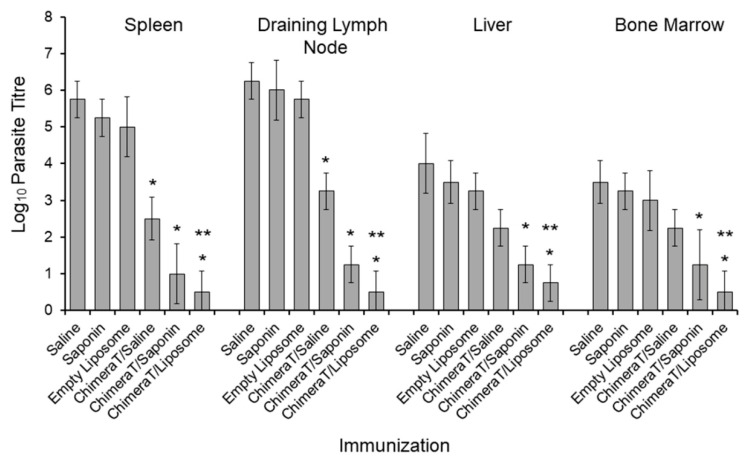
Evaluation of the parasite burden by a limiting dilution technique. BALB/c mice (n = 8 per group) were immunized with ChimeraT/saline, ChimeraT/saponin, and ChimeraT/liposome and controls received saline, saponin, or empty liposome. Thirty days after the last vaccine dose, mice were challenged with 10^7^ *L. infantum* stationary promastigotes, and 45 days after infection, their spleens, livers, draining lymph nodes, and bone marrows were collected for quantifying the parasite loads. The bars indicate the mean ± standard deviations of the groups. (*) indicates a significant difference compared to the control saline, saponin, and empty liposome groups (*p* < 0.0001). (**) indicates a statistically significant difference compared to the ChimeraT/saline group (*p* < 0.0001).

A limiting dilution technique quantified parasitism in the spleen and draining lymph nodes of control mice (saline, saponin, empty liposome) as 10^5^–10^6^ *Leishmania* and lower in the liver and bone marrow at ~10^3^–10^4^ *Leishmania* (Figure 5). Vaccination with ChimeraT/saline reduced the parasite load significantly in the spleen and draining lymph nodes (to ~320–1800 parasites, >98% reduction), and in the liver and bone marrow (to ~200 parasites, >94% reduction) (Figure 5). Even higher reductions in parasite load were shown in mice vaccinated with ChimeraT/saponin and ChimeraT/liposome, with ≤17 organisms recorded in all organs (>99.5% reduction compared to controls) (Figure 5).

**Figure 6:**
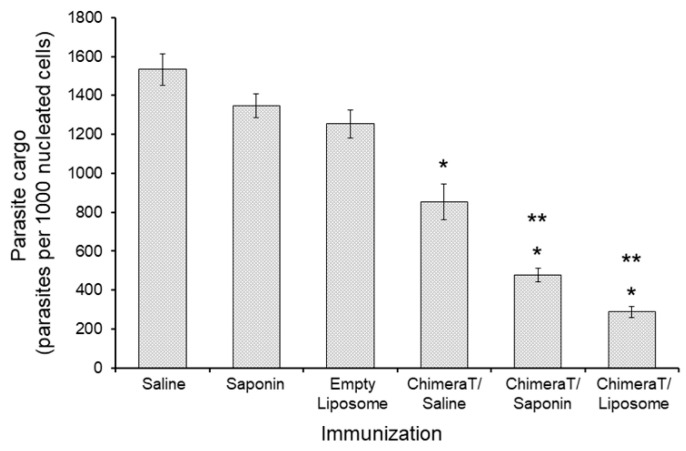
Evaluation of the parasite burden by RT-PCR. BALB/c mice (n = 8 per group) were immunized with ChimeraT/saline, ChimeraT/saponin, and ChimeraT/liposome and controls received saline, saponin, or empty liposome. Thirty days after the last vaccine dose, mice were challenged with 10^7^ *L. infantum* stationary promastigotes, and 45 days after infection, their spleens were collected. Parasite load was estimated by RT-PCR. The bars indicate the mean ± standard deviations of the groups. (*) indicates significant difference compared to the control saline, saponin, and empty liposome groups (*p* < 0.0001). (**) indicates a statistically significant difference compared to the ChimeraT/saline group (*p* < 0.0001).

Reductions in the parasite burden quantified by the limiting dilution technique were confirmed by RT-PCR. Vaccination with ChimeraT/liposome and ChimeraT/saponin reduced parasitism by 77.0% and 65.0%, respectively, i.e., to ~290 and ~480 parasites per 1000 nucleated cells, respectively, when compared with ~1260 and ~1350 parasites per nucleated cells found in the control saponin and empty liposome, respectively (Figure 6). Although immunization with ChimeraT/saline also reduced parasitism significantly by 45.0% (~860 parasites) when compared to the control saline group (~1500 parasites), this level of reduction was significantly lower than that observed for the mice immunized with ChimeraT/liposome or ChimeraT/saponin.

### 3.4. Post-Therapy Serological Follow-up

**Figure 7:**
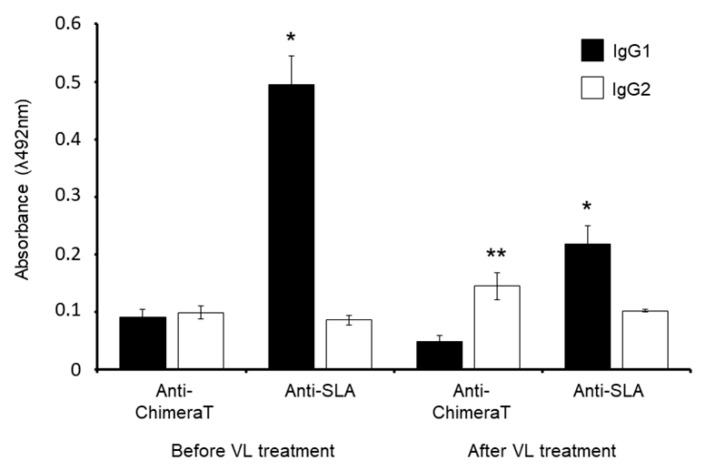
Production of IgG1 and IgG2 subclass antibodies in patients with VL, before and after treatment. Sera were collected from patients with VL, before and after treatment (n = 8 per group), and the anti-ChimeraT and anti-SLA IgG1 and IgG2 subclass levels were examined qualitatively by ELISA. The bars indicate the mean ± standard deviations of the groups. (*) indicates a significant difference compared to the anti-SLA IgG2 level (*p* < 0.0001). (**) indicates a statistically significant difference compared to the anti-ChimeraT IgG1 level (*p* < 0.0001).

Anti-ChimeraT protein and anti-parasite IgG1 and IgG2 isotype levels were evaluated qualitatively in sera from VL patients before and after treatment. Using ChimeraT as the ELISA detection antigen, patients before treatment showed similar levels of IgG1 and IgG2 antibodies (ratios of IgG2/IgG1 and IgG1/IgG2 of ~1.0) (Figure 7, Table 3). Treatment resulted in an increase in IgG2 antibodies to ChimeraT and the IgG2/IgG1 ratio was increased to 3-fold. VL patients before treatment had high levels of IgG1 antibodies to SLA, with an IgG1/IgG2 ratio of ~6, which fell significantly following treatment by ~60% (IgG1/IgG2 ratio of ~2). The levels of IgG2 antibodies to SLA remained unchanged before and after treatment (Figure 7).

## 4. Discussion

The key finding from the current study was that immunization with ChimeraT/liposome generated a significant polarized Th1-type response and protection against *Leishmania* infection of mice. Immunization with ChimeraT/liposome induced significant reductions in the parasite load in mouse organs. It also induced murine anti-ChimeraT protein and an anti-parasite IFN-γ, IL-12, GM-CSF, and IgG2a responses that were augmented significantly after *L. infantum* infection. Notably, this liposomal formulation performed similarly to the saponin adjuvant formulation that has been used successfully with other recombinant *Leishmania* proteins. However, saponin is no longer recommended for human use and the ability of the liposomal formulation to replace it brings closer the possibility of a human trial. Notably, the liposomes prepared in this study are like other non-toxic and biodegradable liposomes, simple to prepare and effective at stimulating the development of a Th1-type response [22,44]. Our ChimeraT/liposome formulation showed a mean diameter of 435.6 nm: this is a near-optimal size for immune system stimulation, since liposomes with an average mean diameter of 500 nm have been reported to efficiently stimulate immune responses [68,69]. However, it is worth noting that immunization with ChimeraT/saline also induced a protective immune response and reduced the parasite load in mouse organs, albeit at a lower level than the ChimeraT-adjuvant formulations. These findings validate our initial in silico studies that identified the most immunogenic T-cell epitopes from the *Leishmania* prohibitin, eukaryotic initiation factor 5a, and hypothetical LiHyp1 and LiHyp2 proteins (Lage et al., manuscript submitted).

In the current study, we performed several in vitro experiments to examine the qualities of the immune response generated by immunization with ChimeraT/liposome in comparison with ChimeraT/saponin. These included evaluating the cellular and humoral responses in cells from immunized mice before and after infection and measuring anti-ChimeraT and anti-parasite IgG antibodies in sera from VL patients, before and after treatment. In human VL, IFN-γ induces NO secretion by *Leishmania*-infected macrophages, which is important for protection against disease [20]. By contrast, cytokines, such as IL-10, deactivate infected cells and contribute to increasing infection [70]. In addition, low levels of IFN-γ and high levels of IL-10 are found in VL patients before treatment, while patients that are treated and cured usually show higher levels of IFN-γ and lower levels of IL-10 [71,72]. For the humoral response, T-cells and produced cytokines are known to stimulate the differential production of antibody isotypes, with IL-10 stimulating the switch from IgG1 to IgG3 production, whereas IFN-γ stimulates the switch to IgG2 production. In our study, we found that ChimeraT/liposome and ChimeraT/saponin induced similar high IFN-γ/IL-10 ratios, suggesting an enhancement of Th-1 type immune activation. Furthermore, ChimeraT/liposome and ChimeraT/saponin induced similar high levels of IFN-γ and of nitrite (NO proxy) and no IL-10, demonstrating the fulfillment of several immune response criteria required of a candidate vaccine. We confirmed that CD4 and CD8 T-cell subtypes produced IFN-γ in the ChimeraT/liposome- and ChimeraT/saponin-immunized mice. Interestingly, mice vaccinated with ChimeraT/saline and then infected with the parasite produced IL-4 and IL-10 cytokines at levels like the control mice, whereas neither cytokine was produced by mice vaccinated with ChimeraT/liposome or ChimeraT/saponin. This suggests that the adjuvant contributes to reducing the production of these cytokines because of the adjuvant-enhanced Th1-type response. Thus, immunization of humans with ChimeraT alone is probably not recommended and exogenous adjuvant will be necessary for full protection.

In our first study (Lage et al., submitted manuscript), we evaluated the lymphoproliferative response and human cytokine production in peripheral blood mononuclear cell cultures from healthy individuals and VL patients, before and after treatment. Results showed T-cell activation, with high lymphoproliferation and IFN-γ production, in response to immune cells stimulated with ChimeraT protein. In the current study, we showed further that a small number of VL patients after treatment had higher levels of circulating anti-protein IgG2 antibodies, compared to IgG1 levels. Tentatively, these results support the cytokine production data in our first study, where we observed an association between IFN-γ and IgG2 in cured and treated patients, whereas high anti-parasite IgG1 antibodies were observed in sera from VL patients before treatment. Indeed, in the current study, the murine and human antibody data were consistent with each other. Thus, the ratio of IgG1/IgG2 antibodies to ChimeraT in sera from control mice after *Leishmania* infection was ~1.0: in human sera, the equivalent would be the VL patients before treatment, where the ratio of IgG1/IgG2 antibodies to ChimeraT was similarly ~1.0. After infection, the same control murine sera showed increased IgG1/IgG2 ratios to SLA of 4–10, and in sera from VL patients before treatment, the IgG1/IgG2 ratio was similar, at ~6.

The limitations of our study should be discussed and form the basis for future investigations of ChimeraT/liposome as a viable candidate VL vaccine. For example, mice could be immunized with ChimeraT and other human-compatible and registered adjuvants, e.g., aluminum hydroxide gel, AF03/AS03/MF59 squalene-based emulsions, and/or with the inclusion of other licensed adjuvants into the liposomes, e.g., monophosphoryl lipid A, in order to increase immunogenicity. Similar to many of our previous experimental vaccine studies in animals [33,73,74,75,76], we used a three-dose immunization schedule with ChimeraT/liposome and future studies would address if fewer vaccinations are necessary to generate protective Th1-type immune responses to develop an optimal vaccination strategy for translation to humans. Such studies also would address the duration of the protective response following ChimeraT/liposome vaccination and whether vaccination induces antigen-specific serum IgE antibodies. Although we and others have used qualitative ELISA to measure serum antibody levels [51,76,77,78,79], quantitative measurements of antibody levels in both mouse and human sera and examination of antigen-specific T-cell proliferative responses in cell culture would further inform the nature of the immune responses. Additionally, the parasite load could be evaluated by examining any histopathological changes in the organs and correlated with data obtained by the limiting dilution technique and RT-PCR. Another limitation of the current study is our pilot study of antibody levels in VL patients examined before and after treatment; ideally, future studies would include much larger patient cohorts.

## 5. Conclusions

The genome of *Leishmania* parasites contains ~8000 genes and many of the encoded proteins are immunogenic in mammalian hosts [80]. Thus, it is probable that vaccines containing multiple antigens and/or epitopes will be needed to protect against VL. In this context, a unique product that contains specific T-cell epitopes from parasite proteins could offer a polarized immune response that protects against parasite infection [28,30]. ChimeraT incorporated into liposomes fulfilled several important criteria for a candidate VL vaccine and deserves consideration for human phase I trials to assess safety and immunogenicity.

## Figures and Tables

**Table 1:** Ratios of IFN-γ/IL-10 cytokines.

Immunization	Ratio IFN-γ/IL-10 in Spleen Cells Stimulated with					
	Before Infection			After Infection		
	Medium	ChimeraT	SLA	Medium	ChimeraT	SLA
**Saline**	0.8	1.0	0.96	0.95	1.38	0.13
**Saponin**	1.03	1.23	1.15	1.04	1.80	0.20
**Empty Liposome**	1.03	1.29	1.01	1.10	1.43	0.21
**ChimeraT/Saline**	1.18	4.28 *	2.31	1.05	8.47 *	0.79
**ChimeraT/Saponin**	1.42	19.96 *	12.21 *	1.42	30.12 *	17.31 *
**ChimeraT/Liposome**	1.41	20.70 *	12.03 *	1.37	32.78 *	19.33 *

Table 1 Caption. The ratio of IFN-γ/IL-10 cytokines was calculated from the peak cytokine levels data shown in Figure 1 for each of the immunization groups. (*) indicates a significant difference compared to the respective control group (*p* < 0.0001).

**Table 2:** Ratios of IgG2a/IgG1 and IgG1/IgG2a antibodies in murine sera.

Immunization	(A) Before Infection				(B) After Infection			
	Anti-ChimeraT		Anti-SLA		Anti-ChimeraT		Anti-SLA	
	IgG2a/ IgG1	IgG1/ IgG2a	IgG2a/ IgG1	IgG1/ IgG2a	IgG2a/ IgG1	IgG1/ IgG2a	IgG2a/ IgG1	IgG1/ IgG2a
**Saline**	1.12	0.89	0.79	1.27	0.92	1.09	0.10	10.10
**Saponin**	1.25	0.80	0.63	1.59	0.82	1.22	0.18	5.60
**Empty Liposome**	1.15	0.89	0.78	1.28	0.83	1.20	0.26	3.85
**ChimeraT/Saline**	1.42	0.7	1.26	0.79	0.95	1.05	0.45	2.22
**ChimeraT/Saponin**	8.84 *	0.11	3.14 *	0.32	6.81 *	0.15	4.19 *	0.24
**ChimeraT/Liposome**	8.58 *	0.12	3.02 *	0.33	10.27 *	0.10	5.29 *	0.19

Table 2 Caption. The peak absorbance data shown in Figure 4 were used to calculate the ratios of IgG2a/IgG1 and the converse IgG1/IgG2a antibodies in murine sera collected before and after infection with *L. infantum.* (*) denotes significant difference compared to the respective control group (*p* < 0.0001).

**Table 3:** Ratios of IgG2/IgG1 and IgG1/IgG2 antibodies in human sera.

	(A) Before VL Treatment		(B) After VL Treatment	
Antigen	IgG2/IgG1	IgG1/IgG2	IgG2/IgG1	IgG1/IgG2
**ChimeraT**	1.08	0.93	2.99	0.33
**SLA**	0.17	5.75	0.47	2.13

Table 3 Caption. The peak absorbance data shown in Figure 7 were used to calculate the ratios of IgG2/IgG1 and the converse IgG1/IgG2 antibodies in sera from patients with VL, before and after treatment.
